# Stress-responsive biomarkers and sexual behaviors among young women in rural South Africa

**DOI:** 10.1093/abm/kaaf060

**Published:** 2025-08-14

**Authors:** Nicole K Kelly, Marie C D Stoner, Sumaya Mall, Kirsten Rowe, Francesc Xavier Gómez-Olivé, Laura Danielle Wagner, Allison E Aiello, Kathleen Kahn, Audrey E Pettifor

**Affiliations:** Division of Infectious Diseases and Global Public Health, Department of Medicine, University of California San Diego, San Diego, CA, 92093, United States; Department of Epidemiology, University of North Carolina at Chapel Hill, Chapel Hill, NC, 27599, United States; Women’s Global Health Imperative, RTI International, Berkeley, CA, 94704, United States; Division of Epidemiology and Biostatistics, School of Public Health, University of the Witwatersrand, Johannesburg, 2193, South Africa; SAMRC/Wits Rural Public Health & Health Transitions Research Unit (Agincourt), School of Public Health, Faculty of Health Sciences, University of the Witwatersrand, Johannesburg, 2193, South Africa; Department of Psychiatry, Faculty of Medicine & Health Sciences, Stellenbosch University, Cape Town, 8000, South Africa; SAMRC/Wits Rural Public Health & Health Transitions Research Unit (Agincourt), School of Public Health, Faculty of Health Sciences, University of the Witwatersrand, Johannesburg, 2193, South Africa; Women’s Global Health Imperative, RTI International, Berkeley, CA, 94704, United States; Department of Epidemiology, Robert N Butler Columbia Aging Center, Mailman School of Public Health, Columbia University, New York, NY, 10032, United States; SAMRC/Wits Rural Public Health & Health Transitions Research Unit (Agincourt), School of Public Health, Faculty of Health Sciences, University of the Witwatersrand, Johannesburg, 2193, South Africa; Department of Epidemiology, University of North Carolina at Chapel Hill, Chapel Hill, NC, 27599, United States; SAMRC/Wits Rural Public Health & Health Transitions Research Unit (Agincourt), School of Public Health, Faculty of Health Sciences, University of the Witwatersrand, Johannesburg, 2193, South Africa

**Keywords:** stress biomarkers, sexual behavior, HIV prevention, South Africa, adolescent girls and young women

## Abstract

**Background:**

Adolescence is a critical period when youth develop their decision-making skills and may engage in their first sexual encounters. Stress during this time can affect decision making; however, limited research has examined the relationship between biological stress correlates and sexual behavior among adolescent girls and young women (AGYW) in high-HIV incidence areas.

**Purpose:**

To examine whether stress-responsive biomarkers are longitudinally associated with sexual behaviors that are predictive of HIV.

**Methods:**

We used data from a cohort of 897 AGYW nested within the HIV Prevention Trials 068 study in rural South Africa. Stress-responsive biomarkers were tested retrospectively from enrollment: C-reactive protein (CRP), herpes simplex virus type-1 (HSV-1) antibody titers, and cytomegalovirus (CMV) antibody titers. We estimated the longitudinal associations between each biomarker (2011-2012) and each behavior throughout follow-up (2011-2019; transactional sex, age-disparate partnerships, multiple partners, and condomless sex).

**Results:**

At enrollment, 25.4% (*n* = 228) had ever had sex, 7.0% (*n* = 63) had >1 partner, 7.9% (*n* = 71) had recent condomless sex, and 3.0% (*n* = 27) reported transactional sex. Compared to low CRP levels, medium and high CRP levels were associated with having an older partner (RR: 1.41 [95% CI, 1.08-1.84]; RR: 1.33 [95% CI, 1.02-1.74], respectively) and with condomless sex (RR: 1.40 [95% CI, 1.10-1.77]; RR: 1.42 [95% CI, 1.12-1.80], respectively).

**Conclusions:**

Higher CRP levels were longitudinally associated with age-disparate relationships and condomless sex. Inflammation may increase AGYW’s engagement in these behaviors; however, future studies should examine whether there is a stress-inflammation-sexual behavior pathway, and if so, evaluate stress-reduction interventions to promote sexual well-being.

## Introduction

Adolescence and young adulthood (ages 15 to 24) is a critical period of human development marked by considerable biologi­cal, cognitive, and socioemotional changes.^1-3^ During this time, young people may have increased desire to experiment and explore with increased impulsivity and changes in risk perception.^2^ These biological, cognitive, and socioemotional changes are also known to influence sexual behaviors,^4^ which is particularly important because many young people engage in their first sexual encounters, experiment with relationships, and develop their sexuality in adolescence.^5-7^

From a developmental lens, it is well documented that sensation seeking peaks in adolescence, whereas self-regulation, the ability to control one’s thoughts, emotions, and behavior to meet long-term goals, is still developing.^8^ Neuroimaging data suggest that adolescents have heightened activation of reward-processing regions, while regions involved in executive function and self-regulation are still maturing throughout adolescence and early adulthood.^9^^,^^10^ These developmental changes can also be impacted by stressful life events during adolescence.^11-13^

The etiology of sexual behavior among AGYW stems from multiple, complex driving forces—both negative and positive—and varies across individuals and contexts. Previous research has highlighted the importance of structural factors (eg, uneven power dynamics, deeply entrenched gender norms),^14-17^ socioeconomic motivations (eg, financial support),^18^ and individual-level drivers (eg, increased autonomy^18^ and pleasure^19^) in the shaping of AGYW’s sexual behavior. Stressful life events (eg, violence) may also play an important role during this critical life stage. Stressful life events encountered during adolescence can result in a more intense and sustained biologic stress response than those encountered in adulthood, which can affect brain development.^20^ There is also evidence that traumatic events (eg, physical or sexual violence) experienced during childhood lead to increased risk taking in youth and adverse health outcomes.^21^ For example, traumatic events can increase depressive symptoms, emotional dysregulation, and impulsivity—all of which have been associated with sexual behaviors that are predictive of HIV acquisition (eg, condomless sex, transactional sex).^22^^,^^23^ Additionally, there is emerging evidence that increased inflammation, which may be a downstream effect of experiencing chronic stressors,^24^ may lead to increased impulsivity and alter decision making.^25^^,^^26^

Ultimately, the effects of stressful life events on sexual behavior during adolescence are complex and likely cannot be explained by a single, unifying theory. However, limited research has been conducted to understand these relationships in high-HIV incidence areas, such as rural South Africa. In our previous work among AGYW in this setting, we observed connections between traumatic events (intimate partner violence) and markers of the biological stress response (eg, inflammation and impaired immune function biomarkers),^27^^,^^28^ and between these biomarkers and HIV acquisition.^29^

Biomarkers of inflammation (eg, C-reactive protein [CRP]) and impaired immune function (eg, cytomegalovirus [CMV] and herpes simplex virus type-1 [HSV-1] antibody titers) are known to elevate in response to stressful life events and psychosocial stressors,^24^^,^^30-32^ such as intimate partner violence^27^^,^^33^ and poverty.^34^ These stressors activate the physiologic stress response which includes the hypothalamic-pituitary-adrenal (HPA) axis, the sympathetic nervous system, and the immune system.^35^ This physiologic response to stress can become dysregulated with acute or chronic stress (ie, high or prolonged exposure to stress) and can lead to high levels of inflammation which can also affect the immune system.^11^ In particular, chronic stress can lead to dysregulated cortisol production which affects the ability of the HPA axis to suppress the inflammatory response and leads to chronic inflammation and immune dysfunction, including high circulating levels of inflammatory cytokines such as CRP.^36^^,^^37^ Due to its effects on the immune system, chronic stress can also cause latent herpes viruses, like CMV and HSV-1, to reactivate, replicate, and increase antibody production.^32^^,^^38^^,^^39^

In this current study, we build on our previous work linking trauma, biomarkers of inflammation and immune function (CRP, CMV, and HSV-1), and HIV^27-29^ to examine whether increased measures of these biomarkers are longitudinally associated with sexual behaviors that are known predictors of HIV acquisition, including transactional sex, age-disparate partnerships, having multiple partners, and condomless sex.

## Methods

### Parent study

This is a secondary analysis of data from a cohort study nested within the HIV Prevention Trials Network (HPTN) 068 study.^40-44^ The parent HPTN 068 study was a randomized controlled trial that examined the effect of a cash transfer, conditional on school attendance, on HIV incidence among AGYW in a rural area of South Africa.^40^ The study was conducted in Bushbuckridge subdistrict in Mpumalanga Province, South Africa—an area with a high HIV prevalence among young women.^45^ HPTN 068 enrolled 2533 young women aged 13 to 20 years who were in school (grades 8-11). Participants were seen annually for up to 3 years during the main trial period (2011-2015). After study completion, participants were followed for 4 more years post-intervention—with visits in 2017 and 2019—for a total of 7 visits over 8 years. All visits included a questionnaire with self-reported information on demographics and sexual behavior administered via Audio Computer-Assisted Self-Interview (ACASI) assessment.^41^^,^^44^ Ethical approval was obtained from the University of Witwatersrand Human Research Ethics Committee Research Ethics Committee and the University of North Carolina at Chapel Hill Institutional Review Board. Informed consent was obtained from all individual participants included in the study. This study was conducted in accordance with the Declaration of Helsinki.

### Current study

We created a cohort nested within HPTN 068 to examine the longitudinal relationship between biological stress correlates (measured at baseline in 2011/2012) and sexual behavior (measured at each study visit in HPTN 068). This “sub-­cohort” was created (*n* = 897) by taking a random sample of participants from HPTN 068 at baseline who were HIV negative, regardless of whether they later tested positive for HIV.^29^ This randomly sampled sub-cohort was designed to be representative of the original study population at enrollment and was used to increase efficiency and minimize costs associated with retrospectively testing biological stress measures from the baseline visit. Methods and results reported for this study follow the Strengthening the Reporting of Observational Studies in Epidemiology (STROBE) guidelines for cohort studies.^46^

### Measures

#### Exposures: stress-responsive biomarkers

The primary exposures were the following stress-responsive biomarkers of inflammation and immune function (hereafter referred to as stress-responsive biomarkers) measured at baseline via dried blood spots (DBS): CRP, CMV, and HSV-1. CRP is a non-specific inflammatory marker that rises in response to various triggers, both acute and chronic, infective and non-infective, including traumatic events.^47^^,^^48^ CMV and HSV-1 are both herpes viruses that can become reactivated due to stressful life events.^32^^,^^39^ We selected these stress-responsive biomarkers because they (1) allow for comparison with previous work^27-29^^,^^49^; (2) are valid, precise, and sensitive; and (3) can be collected via DBS in low resource areas.

CRP levels (mg/L) were measured using the CRP Enzyme-Linked Immunosorbent Assay (ELISA) from Immunodiagnostik (IDK). Values that were out of range (below detectable level) were coded as 0.015 (the lower limit of the test) and extreme outliers (over 40) were removed. We then created a 3-level ordinal CRP variable based on tertiles (1 = lowest tertile [0.015-0.848], 2 = medium [0.849-3.236], 3 = highest tertile [≥3.327]). Tertiles were selected to be consistent with the herpesvirus biomarker operationalizations (detailed below) and because the CRP distribution was highly right skewed. In previous work, we considered using a continuous CRP measure but ultimately determined that this continuous measure had limited clinical importance and interpretability.^27^ We did not use CRP categories established by the Centers for Disease Control and Prevention and the American Heart Association, as these thresholds were validated in a sample of adults of European and American-European ancestry,^50^ and CRP values are known to differ by age and race.^51^^,^^52^

The HSV-1 and CMV measures in this study included primary infection and optical density (OD) levels, where OD levels for Immunoglobulin G (IgG) tests approximately corresponded to antibody titer (low and positive OD indicated low antibody titer; high OD indicated high antibody titer). CMV and HSV-1 OD levels were measured using Trinity Biotech Captia™ CMV IgG ELISA and Trinity Biotech Captia™ HSV-1 IgG kit ELISA, respectively. Similar to prior analyses,^27-29^ we coded the CMV and HSV-1 variables using 4 ordinal categories: seronegative individuals (0) and OD levels divided into tertiles (1 = low, 2 = medium, and 3 = high, with cutoffs at 0.232 and 0.465 for HSV-1 and at 1.310 and 1.897 for CMV). Equivocal results were then coded as low OD levels.

#### Outcomes: sexual behaviors

The primary outcomes were sexual behaviors that were self-­reported at each visit and included: (1) transactional sex (sex with a male partner in exchange for money or gifts in the past year [yes/no]),^53^ (2) age-disparate partnership (having a male partner who is 5 or more years older [yes/no]),^54^ (3) condomless sex (in the past 3 months [yes/no]),^40^ and (4) having more than 2 sexual partners in the past year (yes/no).^40^^,^^41^^,^^44^^,^^55^ We examined these outcomes as repeated measures at each study visit and as longitudinal trajectories (detailed in the “Statistical analysis” section).

#### Covariates

**Figure 1.**
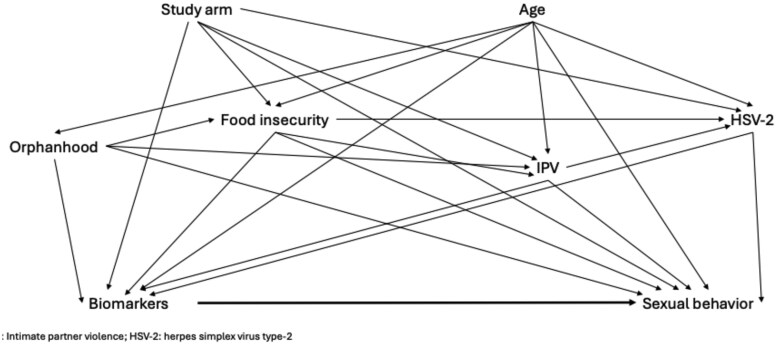
Directed acyclic graph of expected causal pathways between stress-responsive biomarkers and sexual behavior among adolescent girls and young women in rural South Africa. Abbreviations: IPV: intimate partner violence; HSV-2: herpes simplex virus type-2.

Potential confounders were (1) measured variables known to affect both the exposure and the outcomes, (2) selected a priori based on other studies using these data^27-29^^,^^49^ and a directed acyclic graph (Figure 1), and (3) aligned with the timing of the primary exposure (ie, measured at baseline). Confounders included age (years), food insecurity (worry about food in the last 12 months [yes/no]), orphanhood before age 18 (loss of a parent [yes/no]), ever experienced physical intimate partner violence (yes/no), intervention arm in the original HPTN 068 study (yes/no), and HSV-2 serostatus (positive/negative). Socioeconomic status was collinear with food insecurity and removed from the final adjustment set. Other stressors that were potential confounders, like adverse childhood experiences, were not measured in this study. HSV-2 was included as a covariate because it is related to coinfections and comorbidities,^39^ and it has been associated with the included biomarkers^49^ as well as with sexual behaviors.^56^ We decided on including HSV-2 as a confounder a priori as it has been included in other manuscripts from the study.^27-29^ HSV-2 testing was done on blood samples at all visits using the HSV-2 IgG ELISA assay (Kalon Biological Ltd, Guildford, UK) with prevalent infection an index cutoff of 1.5.^40^^,^^42^^,^^43^ Socioeconomic status (SES) was measured using a wealth index, where AGYW were classified as being of low, middle-low, middle-high, or high SES based on assets.

### Statistical analysis

We used log binomial regression to estimate the longitudinal association between each stress-responsive biomarker at baseline and each sexual behavior throughout the study period. Generalized estimating equations (GEEs) with an exchangeable correlation matrix were used to account for repeated measures over the study period. From each model, we report a risk ratio (RR) and corresponding 95% confidence interval (CI).

As an alternative specification of the sexual behavior outcomes, we also used group-based trajectory modelling (GBTM) to identify longitudinal trajectories of each sexual behavior. GBTM facilitates identification of latent groups of individuals whose outcomes of interest follow a similar trajectory over time.^57^^,^^58^ We first fit a series of unconditional trajectory models for each sexual behavior to identify the most appropriate number of trajectory groups, considering up to 4 groups. Model fit was determined using the following criteria: (1) how groups corresponded with substantive literature about trends in sexual behaviors over time; (2) average posterior class probabilities of class membership; (3) Akaike Information Criteria (AIC; smaller values indicating better fit); (4) Bayesian Information Criteria (BIC; smaller values indicating better fit); (5) entropy (values closer to one indicate accurate classifications); (6) percentage and size of smallest trajectory group; and (7) the log likelihood of each model.^59^ After the best-fitting number of trajectories was selected, we then considered constant, linear, quadratic, and cubic specifications for each trajectory group, selecting a final model through visual inspection of the data and using fit statistics described above for fitting individual group trajectories.

Lastly, we examined how the baseline stress-responsive biomarkers related to these longitudinal trajectories of sexual behavior throughout the study period. We did this by using log binomial regression to estimate the association between each biomarker and trajectory group for each sexual behavior. The trajectory group characterized by a lower prevalence of that sexual behavior outcome was chosen as the referent group for all models. Analyses were done using Stata version 16; trajectory models were estimated using the *traj* command.

## Results

### Demographic characteristics and sexual behaviors

A total of 897 participants were randomly sampled for this nested cohort study. At enrollment, the median age was 15 (IQR: 14, 17), 34.9% (*n* = 313) were food insecure, 21.2% (*n* = 190) had a mother or father die before age 18, 50.3% (*n* = 451) were randomized to the intervention arm, and 17.3% (*n* = 155) had ever experienced physical intimate partner violence (Table 1). All participants identified as Black African women or girls; 28% were of low SES, 28.3% were of middle-low, 22.5% were of middle-high, and 21.1% were of high SES. Demographic characteristics of the sub-cohort were representative of those for the entire HPTN 068 study.^40^ When examining sexual behaviors at enrollment, 25.4% (*n* = 228) had ever had sex, 7.0% (*n* = 63) had more than one partner in the past 12 months, 7.9% (*n* = 71) had condomless sex in the past 3 months, and 3.0% (*n* = 27) reported transactional sex in the past 12 months (Table 2).

**Table 1.** Baseline demographic characteristics in a cohort of adolescent girls and young women in rural South Africa (*n* = 897).

	Total (*n* = 897)	
	*N*/median	%/IQR
**Age, median IQR**	15	14, 17
**CRP (mg/L), median IQR**	1.55	0.54, 4.72
**CMV (OD),^a^ median IQR**	1.53	1.22, 2.15
**HSV (OD),^a^ median IQR**	0.55	0.45, 0.67
**CRP levels**		
Low	299	33.3
Middle	288	32.1
High	276	30.8
Missing^b^	34	3.8
**CMV serostatus and OD levels**		
Negative	155	17.3
Positive and low OD levels	249	27.8
Positive and middle OD levels	251	28.0
Positive and high OD levels	242	27.0
**HSV-1 serostatus and OD levels**		
Negative	484	54.0
Positive and low OD levels	160	17.8
Positive and middle OD levels	130	14.5
Positive and high OD levels	123	13.7
**Intervention arm**		
Control	446	49.7
Intervention	451	50.3
**Grade enrolled**		
9	229	25.5
10	253	28.2
11	233	26.0
12	182	20.3
**Ever pregnant**		
No	809	90.2
Yes	83	9.3
Missing	5	0.6
**Alcohol more than once a month**		
No	876	97.7
Yes	20	2.2
Missing	1	0.1
**If mother or father died when age <18**		
No	702	78.3
Yes	190	21.2
Missing	5	0.6
**Ever any IPV**		
No	725	80.8
Yes	155	17.3
Missing	17	1.9
**IPV in the past 12 months**		
No	788	87.8
Yes	92	10.3
Missing	17	1.9
**Depression** ^c^		
No	710	79.2
Yes	150	16.7
Missing	37	4.1
**Food insecurity**		
No	577	64.3
Yes	313	34.9
Missing	7	0.8
**Wealth** ^d^		
Low	251	28.0
Middle-low	254	28.3
Middle-high	202	22.5
High	189	21.1
Missing	1	0.1

Abbreviations: CMV, cytomegalovirus; CRP, C-reactive protein; HSV-1, herpes simplex virus type-1; IPV, intimate partner violence; OD, optical density.

a Median CMV and HSV-1 OD values were restricted to seropositive participants; median CRP levels were based on the entire sample.

b Missing CRP values were those that were extreme outliers that did not have biologically plausible values.

c Measured using the Children’s Depression Inventory (≤7).

d Based on a household asset index.

**Table 2.** Sexual behaviors at baseline in a cohort of adolescent girls and young women in rural South Africa (*n* = 897).

	Total (*n* = 897)	
	No.	%
**Ever had sex**		
No	667	74.4
Yes	228	25.4
Missing	2	0.2
**Coital debut before age of 15**		
No	832	92.8
Yes	61	6.8
Missing	4	0.4
**Age at coital debut, median IQR (** *N* = **228)** ^a^	15	14, 16
**Had sexual partners in past 3 months**		
No	688	76.7
Yes	201	22.4
Missing	8	0.9
**Had sexual partners in past 12 months**		
No	656	73.1
Yes	228	25.4
Missing	13	1.4
**Older partner ≥5 years older** ^b^		
No	831	92.6
Yes	51	5.7
Missing	15	1.7
**Transactional sex**		
No	814	90.7
Yes	27	3.0
Missing	56	6.2
**Condomless sex in past 3 months**		
No	820	91.4
Yes	71	7.9
Missing	6	0.7
**Number of partners in past 12 months**		
0	656	73.1
1	165	18.4
>1	63	7.0
Missing	13	1.4
**CRP level, median IQR**	1.66	0.56, 4.88
**CMV OD levels in seropositive, median IQR**	1.53	1.22, 2.15
**HSV-1 OD levels in seropositive, median IQR**	0.55	0.45, 0.68

a All measures were asked in the full sample (*N* = 897) unless otherwise specified.

b Sexual or non-sexual partnership.

### Stress-responsive biomarkers and sexual behavior

Compared to low levels, medium and high CRP levels were associated with having an age-disparate relationship over the study period, adjusting for covariates (medium RR: 1.31, 95% CI, 1.04-1.66; high RR: 1.33, 95% CI, 1.04-1.68; Table 3). Similarly, medium (RR: 1.30; 95% CI, 1.05-1.59) and high CRP levels (RR: 1.33; 95% CI, 1.08-1.64) were associated with having any condomless sex over the study period, adjusting for covariates. Compared to being HSV-1 seronegative, medium HSV-1 OD levels were associated with a greater likelihood of having more than one partner in the past 12 months (OR 1.46; 95% CI, 1.07-1.99). We did not find statistically significant associations between CMV with any sexual behavior outcomes, after adjusting for covariates.

**Table 3.** Risk ratios (RRs) and 95% confidence intervals (CIs) for the associations between stress-responsive biomarkers at enrollment and sexual health outcomes over the study period (*n* = 897).

	Older partner		Transactional sex		More than 1 partner in last 12 months		Any condomless sex last 3 months	
	Unadjusted	Adjusted^a^	Unadjusted	Adjusted	Unadjusted	Adjusted	Unadjusted	Adjusted
	RR (95% CI)	RR (95% CI)	RR (95% CI)	RR (95% CI)	RR (95% CI)	RR (95% CI)	RR (95% CI)	RR (95% CI)
**CRP levels**								
Low	1	1	1	1	1	1	1	1
Middle	**1.40 (1.11-1.77)** ^b^	**1.31 (1.04-1.66)**	1.12 (0.84-1.51)	0.96 (0.72-1.28)	1.10 (0.82-1.48)	0.98 (0.73-1.32)	**1.46 (1.18-1.80)**	**1.30 (1.05-1.59)**
High	**1.50 (1.19-1.89)**	**1.33 (1.04-1.68)**	**1.44 (1.09-1.91)**	1.08 (0.81-1.44)	1.19 (0.89-1.60)	1.02 (0.75-1.38)	**1.65 (1.33-2.03)**	**1.33 (1.08-1.64)**
**CMV levels**								
Negative	1	1	1	1	1	1	1	1
Low	0.89 (0.67-1.18)	0.86 (0.65-1.14)	0.99 (0.70-1.39)	0.99 (0.71-1.37)	1.05 (0.72-1.53)	1.00 (0.69-1.45)	0.98 (0.77-1.25)	0.94 (0.75-1.19)
Middle	1.20 (0.82-1.57)	1.09 (0.84-1.42)	1.06 (0.76-1.49)	0.95 (0.68-1.32)	1.39 (0.97-1.99)	1.24 (0.87-1.77)	1.06 (0.83-1.35)	0.94 (0.74-1.17)
High	0.91 (0.68-1.20)	0.92 (0.70-1.22)	0.92 (0.65-1.31)	0.97 (0.69-1.37)	1.05 (0.72-1.53)	1.12 (0.77-1.63)	0.85 (0.66-1.09)	0.84 (0.66-1.07)
**HSV-1 levels**								
Negative	1	1	1	1	1	1	1	1
Low	0.98 (0.78-1.26)	0.95 (0.74-1.22)	0.70 (0.50-0.98)	0.67 (0.48-0.94)	0.94 (0.68-1.32)	0.91 (0.65-1.26)	1.01 (0.81-1.25)	0.98 (0.79-1.20)
Middle	1.08 (0.84-1.40)	1.17 (0.90-1.51)	0.84 (0.60-1.18)	0.88 (0.63-1.23)	1.28 (0.93-1.76)	**1.46 (1.07-1.99)**	0.86 (0.66-1.11)	0.88 (0.69-1.13)
High	1.00 (0.76-1.32)	1.03 (0.78-1.35)	0.91 (0.65-1.28)	0.90 (0.65-1.23)	1.02 (0.72-1.46)	1.09 (0.76-1.54)	1.04 (0.82-1.33)	1.02 (0.81-1.29)

Abbreviations: CMV, cytomegalovirus; CRP, C-reactive protein; HSV-1, herpes simplex virus type-1.

a Covariates at enrollment include randomization arm, age, food insecurity, orphanhood, ever experienced intimate partner violence, and herpes simplex virus type-2 serostatus.

b Bolded values = *p* < 0.05.

**Table 4.** Associations between stress-responsive biomarkers at enrollment and trajectories of sexual health outcomes among the sub-cohort (*n* = 897).^a^

	Older partner (low trajectory vs high)	Transactional sex (low trajectory vs high)	Any condomless sex last 3 months	More than 1 partner in last 12 months
	RR (95% CI)	RR (95% CI)	RR (95% CI)	RR (95% CI)
**CRP levels**				
Low	1	1	1	1
Middle	**1.59 (1.12-2.27)** ^b^	1.10 (0.79-1.54)	1.17 (0.55-2.47)	0.60 (0.32-1.13)
High	1.42 (0.98-2.06)	1.13 (0.80-1.58)	1.90 (0.94-3.83)	0.86 (0.48-1.55)
**CMV levels**				
Negative	1	1	1	1
Low	0.92 (0.59-1.49)	0.89 (0.60-1.32)	1.13 (0.50-2.54)	0.86 (0.40-1.86)
Middle	1.3 7 (0.92-2.06)	0.96 (0.66-1.40)	0.96 (0.42-2.21)	1.04 (0.50-2.18)
High	1.14 (0.74-1.77)	0.99 (0.67-1.47)	1.00 (0.43-2.36)	1.19 (0.56-2.51)
**HSV-1 levels**				
Negative	1	1	1	1
Low	1.00 (0.69-1.43)	0.94 (0.65-1.34)	1.02 (0.51-2.04)	1.16 (0.61-2.19)
Middle	1.11 (0.75-1.64)	0.90 (0.60-1.36)	0.64 (0.25-1.66)	1.62 (0.86-3.05)
High	0.94 (0.61-1.46)	1.01 (0.68-1.48)	0.97 (0.42-2.20)	0.88 (0.38-2.06)

Abbreviations: CMV, cytomegalovirus; CRP, C-reactive protein; HSV-1, herpes simplex virus type.

a Covariates at enrollment include randomization arm, age, food insecurity, orphanhood, ever experienced intimate partner violence, and herpes simplex virus type-2 serostatus.

b Bolded values = *p* < 0.05

### Sexual behavior trajectories

**Figure 2.**
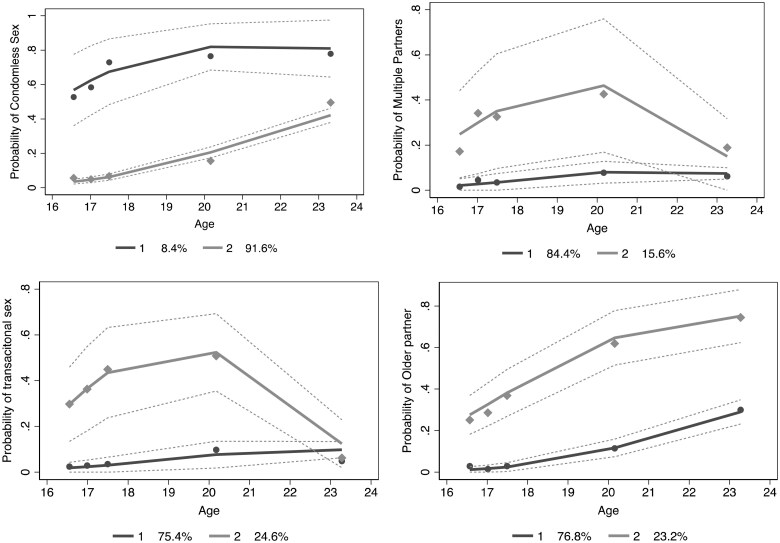
Trajectories of sexual behaviors over time among a cohort of adolescent girls and young women in rural South Africa (*n* = 897).

When examining trajectories of the 4 sexual behaviors, we found that all outcomes had one group with a lower probability of that outcome over time (lower trajectory group) and a second group with a higher probability of the outcome over time (higher trajectory group; Figure 2). However, the shape of these trajectory curves varied across sexual behaviors. For condomless sex and having an older partner, the probability of the outcome increased in both the low and high groups over the study period. For transactional sex and multiple partners, the probability of the outcome decreased in both the low and high groups over the study period, although this change was much smaller in the low group.

Compared to low CRP levels, medium CRP levels (RR: 1.59; 95% CI, 1.12-2.27) were associated with belonging to the group with a higher probability of age-disparate relationships over the study period, adjusting other covariates (Table 4). None of the other associations were statistically significant at an alpha of 0.05, although those with increased CRP levels (medium RR: 1.10; 95% CI, 0.79-15.54; high RR: 1.13; 95% CI 0.80-1.58) were non-significantly associated with belonging to the group with a higher incidence of any recent condomless sex. We did not observe dose-response relationships between higher biomarker levels and stronger associations with sexual outcomes, nor did we identify statistically significant relationships between any biomarkers and groups with higher probabilities of transactional sex or having more than one partner in the past 12 months (Table 4).

## Discussion

In a cohort of AGYW in rural South Africa, having higher CRP levels in early and middle adolescence was longitudinally associated with engaging in age-disparate partnerships and condomless sex throughout adolescence and into early adulthood. We similarly observed a relationship between CRP and age-disparate partnerships when using GBTM to model sexual behaviors over time. CMV levels were not longitudinally associated with any of the sexual behavior outcomes, and HSV-1 was only associated with having multiple sexual partners. CRP is a general marker of systemic inflammation that can increase in response to stress due to traumatic event exposure or other biological processes.^47^^,^^48^ The findings from this study suggest that increased inflammation, which is evidenced by CRP, could play a role in AGYW’s sexual behavior, including entering a partnership with an older man or engaging in condomless sex. However, future research is needed to determine whether there is a pathway from stress to sexual behavior through inflammation,^60^ or if inflammation increases stress appraisals by altering central nervous system functioning.^61^ Pinpointing these pathways is crucial to creating tailored interventions that support AGYW and promote their overall sexual well-being.

AGYW in this population are likely balancing many evolving and competing desires, needs, and life events, including those that may be stressful, while also developing their decision-making skills and navigating their first sexual encounters.^2^^,^^62^ There is evidence that the biologic response to stress can lead to increased impulsivity and risk taking in youth,^21^ which could explain the association between higher CRP levels, age-disparate partnerships, and condomless sex. Alternatively, inflammation may predispose AGYW to depressive symptoms by depleting serotonin precursors and increasing blood-brain barrier permeability to inflammatory cytokines resulting in dopamine depletion and increased glutamate, both of which may lead to anhedonia.^63^ Anhedonia may lead to increased sensation seeking to experience reward or pleasure. While future work to understand the biological pathways from inflammation to sexual behavior is essential, it is also critical to highlight that these sexual behaviors may be a mediating pathway between biological stress and HIV among AGYW, particularly given the observed relationships in this analysis between CRP, age-disparate relationships, and condomless sex. This pathway is further supported by our previous research which found that elevated levels of stress-responsive biomarkers (ie, CRP, HSV-1, and CMV) were associated with HIV acquisition in the same population of rural South African AGYW.^29^

The effects of stressful life events, such as traumatic events, on young people’s health are wide-reaching and potentially long-lasting.^64^ This study provides evidence that early life biological stress, possibly due to adverse childhood experiences and traumatic events, may affect sexual behavior or that AGYW may engage in certain sexual behaviors as an adaptive trauma response. In our previous work, we observed associations between intimate partner violence and some of these stress-responsive biomarkers (ie, CRP).^27^^,^^28^ Being in a violent relationship may put the body in a fight-flight-freeze state and perpetuate repetitive or continuous elevations in stress-responsive biomarkers.^48^^,^^65^ The diagnostic criteria for post-traumatic stress disorder include “reckless or self-destructive behavior” and “irritable behavior or angry outbursts (with little or no provocation), typically expressed as verbal or physical aggression towards people or objects,” which could be a perpetuating factor in the ongoing cycle of intimate partner violence-to-stress within a relationship.^66^ Furthermore, age-disparate partnerships have been associated with an increased risk of intimate partner violence,^67^ so this sexual behavior may mediate the relationship between stressful life events and violence and be a future area of intervention.

Additionally, public health research primarily frames the sexual behaviors reported in this study as “risky,” and most research only attributes these behaviors to AGYW being disempowered.^68^ Yet, this narrative does not reflect the reality of the nuanced array of factors driving AGYW’s sexual decision-making in Southern Africa. For example, some AGYW may engage in age-disparate partnerships as an expression of their own agency or based on their own values and priorities.^69^ These decisions are not necessarily impulsive nor reflective of poor self-regulation. Thus, future research is needed to examine whether factors influencing decision-making, like impaired executive function, mediate the relationship between biological stress and these sexual behaviors and to better understand AGYW’s motivations behind these sexual behaviors. More work is also needed to understand interventions to support AGYW in coping with stressful life events while balancing competing motivations surrounding sex—such as informed choice, pleasure, and risk taking—that are a normative and healthy part of sexual development.^70^

Overall, this study was strengthened by its design, measurement of stress-responsive biomarkers, and use of repeated measures. The nested cohort design allowed us to create a representative sample of the parent study, employ rigorous biological testing, and simultaneously minimize costs. Furthermore, there is extensive literature showing that the biomarkers in our study (CMV, CRP, and HSV-1) are valid and are associated with stress and health outcomes.^71^^,^^72^ Using repeated measures of sexual behavior allowed us to examine these relationships longitudinally during a critical developmental period for AGYW. However, this study contains several limitations, including that stress-responsive biomarkers were only measured at one time point. Additionally, CRP is a generic measure of inflammation, and HSV-1 and CMV are related to viral infection making it hard to pinpoint causality. Yet, the benefits of using these measures (sensitivity, validity, practicality) outweighed their limitations. The use of 3 different biomarkers and several behavioral outcomes does raise the issue of multiple comparisons. However, we previously observed the strongest associations between trajectories of intimate partner violence and CRP in this same population,^28^ strengthening the likelihood that this is a true association rather than a false positive.

We also used GBTM in this study to examine longitudinal behavior patterns, but this approach is data driven and, therefore, the trajectories that emerge are dependent on duration of the study and sample size. Therefore, we may have identified a larger number of trajectories with a larger sample. However, our models showed good model fit and the patterns that we observed were similar to those seen in other studies including analyses using the same data to fit trajectories.^28^^,^^73^ Lastly, this study may not be generalizable to AGYW in other rural southern African contexts, as the parent study was restricted to AGYW who were enrolled in school.

## Conclusion

In a sample of AGYW in rural South Africa, increased CRP levels at baseline were longitudinally associated with having an older male partner and engaging in condomless sex. AGYW have many reasons for engaging in sexual behaviors and are learning to navigate the risks and benefits of these behaviors during this key developmental period. Our findings suggest that inflammation may affect AGYW’s sexual behavior and decision making, possibly due to stressful life events, alterations in central nervous system function, or both. Future work should evaluate these pathways longitudinally to more fully understand these relationships and to inform interventions that support AGYW in balancing their evolving needs and desires as a normative and healthy part of sexual development.

## Transparency statements

(1) *Study registration*: This study was not formally registered, but the original parent study was pre-registered at ClinicalTrials.gov (https://www.clinicaltrials.gov/study/NCT01233531). (2) *Analytic plan pre-registration*: The analysis plan was not formally pre-registered. (3) *Data availability*: Most de-identified data from this study are available in a public archive: https://dataverse.harvard.edu/dataverse/HPTN068. Additional de-­identified data from this study will be made available (as allowable according to institutional IRB standards) by emailing the corresponding author. (4) *Analytic code availability*: Analytic code used to conduct the analyses presented in this study are not available in a public archive. They may be available by emailing the corresponding author. (5) *Materials availability*: Some of the materials used to conduct the study are presented in a public archive: https://www.hptn.org/research/studies/hptn-068.
